# Amyloid Protein-Induced Remodeling of Morphometry and Nanomechanics in Human Platelets

**DOI:** 10.3390/biomedicines13123104

**Published:** 2025-12-16

**Authors:** Tonya D. Andreeva, Svetla Todinova, Ariana Langari, Velichka Strijkova, Vesela Katrova, Stefka G. Taneva

**Affiliations:** 1 Faculty Life Sciences, Reutlingen University, Alteburgstraße 150, 72762 Reutlingen, Germany; 2 Institute of Biophysics and Biomedical Engineering, Bulgarian Academy of Sciences, “Acad. G. Bonchev” Str. 21, 1113 Sofia, Bulgaria; todinova@abv.bg (S.T.); arianalangari@abv.bg (A.L.); 3 Institute of Optical Materials and Technologies “Acad. Yordan Malinovski”, Bulgarian Academy of Sciences, “Acad. G. Bonchev” Str. 109, 1113 Sofia, Bulgaria; vily@iomt.bas.bg (V.S.); vesela.lozanova@abv.bg (V.K.)

**Keywords:** platelets, neurodegenerative diseases, amyloid β peptide, α-synuclein, platelet morphometry, platelet nanomechanics

## Abstract

**Background/Objectives**: The accumulation of specific amyloid proteins and peptides in the human brain is a hallmark of neurodegenerative disorders such as Alzheimer’s disease (AD) and Parkinson’s disease (PD). Beyond the central nervous system, circulating peripheral blood cells are also exposed to these pathological proteins, which may contribute to the systemic disease manifestation. Human platelets (PLTs) were used as an in vitro model to investigate the impacts of amyloid Aβ1-42 peptide oligomers (Aβ42) and on-pathway α-synuclein (α-syn), two key amyloids implicated in AD and PD, on platelet biophysical properties. **Methods**: Using atomic force microscopy, imaging and force–distance modes, we analyzed changes in surface nanostructure, morphometric and nanomechanical signatures of PLTs, derived from healthy donors, following exposure to increasing concentrations of Aβ42 and α-syn. **Results**: Our findings show that platelet activation progresses with increasing amyloid concentration, characterized by cytoskeletal remodeling (filopodia-to-pseudopodia and lamellipodia transformation). While Aβ42 causes progressive decrease in the platelet membrane roughness, α-syn exhibits a biphasic effect—initial smoothing followed by a pronounced increase in the roughness at high concentrations. Both amyloids induce substantial increase in membrane stiffness (Young’s modulus). **Conclusions**: The changes in PLTs’ biophysical properties closely resemble the previously observed modification in PLTs derived from AD and PD patients, suggesting that amyloid proteins’ interactions with PLTs may contribute to their dysfunction. Our results underscore the potential of platelets as peripheral indicators of neurodegeneration and point to their role in the systemic pathology of amyloid-associated diseases.

## 1. Introduction

The formation and progressive accumulation of misfolded protein aggregates in the human brain are hallmarks of neurodegenerative disorders (NDDs), with these proteins capable of self-assembly and formation of heteroaggregates in the brain, peripheral blood cells, and cellular models [1,2,3,4,5,6,7,8,9]. The abnormal behavior and aggregation of amyloid β-peptides (Aβ) into extracellular amyloid plaques are primarily associated with Alzheimer’s disease (AD) [10,11,12]. Similarly, the misfolding and aggregation of the protein α-synuclein (α-syn) are linked to “synucleinopathies”, particularly Parkinson’s disease (PD) [13,14,15,16].

Importantly, amyloids are not confined to the brain: Aβ can exit the brain by crossing the blood–brain barrier (BBB), circulate within the bloodstream, and interact with peripheral blood cells, including platelets (PLTs) and red blood cells (RBCs) [17], and can enter the brain and contribute to amyloid deposition [18,19]. Platelets are a primary peripheral source of Aβ, accounting for approximately 90% of circulating Aβ peptides [20]. Upon activation, platelets release Aβ, which can influence vascular function and potentially contribute to cerebral amyloid accumulation [20,21,22]. Aβ peptides have been detected within human RBCs, where they may induce oxidative stress and impair the cells’ oxygen-delivery capacity, suggesting a role in systemic vascular dysfunction associated with neurodegenerative diseases [7]. Studies have shown that Aβ peptides in plasma exist in a dynamic equilibrium with Aβ levels in the brain. Consequently, plasma Aβ levels are considered potential indicators of Aβ accumulation in the brain [23,24,25]. Elevated plasma Aβ levels have been observed in individuals with cognitive impairment compared to healthy subjects [26]. However, it remains unclear whether plasma Aβ levels alone are sufficient to reliably distinguish AD patients from healthy subjects, as results across studies are somewhat inconsistent. Similarly, α-synuclein (α-syn) has been detected in plasma, and significantly higher plasma concentrations of α-syn have been recorded in patients with PD compared to healthy individuals [27,28,29,30,31,32,33].

For decades, PLTs have been considered a convenient peripheral model for studying AD pathophysiology [34,35,36] and as a promising source of biomarkers [36,37,38]. PLTs share several key biochemical properties with neurons implicated in AD, including increased β-secretase activity and secretion of amyloid proteins [37,39,40]. Moreover, they represent the major peripheral source of amyloid precursor protein (APP) in blood plasma [41,42,43,44]. Upon activation, PLTs undergo proteolytic cleavage of APP that occurs both at the PLT cell membrane and in the extracellular compartments, leading to release of significant quantities of Aβ peptides that closely resemble those found in the senile plaques of AD patients [35,44,45,46]. The isoforms of Aβ peptide released by PLTs mirror those deposited in the brains of individuals with AD [47]. However, the precise origin of circulating Aβ peptides, whether primarily secreted by PLTs or transported across the blood–brain barrier from the central nervous system, remains a subject of ongoing debate [48,49].

The pathological intrinsically disordered protein α-syn is expressed both in neurons and in PLTs [44,50,51,52,53]. Flow cytometric analysis using monoclonal antibodies targeting different α-syn regions (specifically the positively charged N-terminus, the hydrophobic non-amyloid β-component (NAC), and the negatively charged C-terminus) has suggested that the structural arrangement of α-syn is similar in both PLTs and neurons [54]. In PD, PLTs have been shown to overexpress α-syn, leading to a variety of pathological alterations, including morphological changes, hyperactivation, increased granule release, excessive production of reactive oxygen species, and mitochondrial dysfunction [55,56,57,58,59,60,61].

Previous investigations have shown significant modifications of the morphological and nanomechanical properties of PLTs derived from patients with NDDs, including AD and PD [60,61]. In the conditions of NDDs, PLTs exhibited changes in surface roughness, stiffness, and activation state compared to healthy individuals, suggesting that such parameters could serve as potential biomarkers [60,61]. While atomic force microscopy (AFM) has been successfully employed to assess the morphology and nanomechanics of platelets in the context of NDDs, there remains a lack of detailed studies specifically examining the direct effect of exogenously applied Aβ peptides or α-syn proteins on healthy platelets using AFM.

The present work aims to understand how the interaction of exogenously applied amyloid proteins affects the physical characteristics of PLTs and to provide insight into specific and/or common modifications of the PLT biophysical signatures induced by the two types of amyloid proteins—Aβ and α-syn. We explore AFM to assess quantitative morphological and mechanical properties of PLTs.

## 2. Materials and Methods

### 2.1. Reagents

Aβ1–42 (human, synthetic) and α-syn were purchased from Kaneka Eurogentec S.A. (LIEGE Science Park, Seraing, Belgium). HFIP (1,1,1,3,3,3-Hexafluoro-2-propanol > 99%) and all reagents were obtained from Sigma-Aldrich (St. Louis, MO, USA).

### 2.2. Preparation of Aβ42 Peptides and α-Syn Samples

Aβ1-42 (Aβ42) peptide oligomers were prepared following the protocol described in [62]. Aβ42 was dissolved in pure HFIP to concentration of 0.57 mg/mL and sonicated for 1 h. Aliquots of Aβ42 were dried under argon and held for 2 h under vacuum. Before being added to PLTs, Aβ42 was dissolved in PBS, passed through 0.45 mm (Millipore, MILLEXs-HV) and 0.22 mm (Millipore, MILLEXs-GV, Burlington, MA, USA) pore filters, and incubated for 1 h at 37 °C [62]. Freshly isolated PLTs were treated with different concentrations of Aβ42 for 15 min and washed twice for 1 min at 150× *g* with PBS to remove the unbound Aβ.

Following the protocol of Grey et al. [63], “on-pathway α-synuclein” samples were obtained by incubating α-syn in PBS buffer, pH 7.4, at 37 °C under continuous shaking for 2 h. Pre-incubated α-syn at different concentrations, up to 20 μM, was added to PLTs for 15 min [63,64], and then the samples were washed twice with PBS for 1 min at 150× *g* to remove the unbound α-syn.

### 2.3. PLT Isolation and Sample Preparation for AFM Experiments

Platelets were isolated from fresh venous blood (10 mL), derived from 14 healthy volunteers (10 females and 4 males, mean age 55.8 ± 2.4), and collected in K2 (EDTA) vacutainers (Becton, Dickinson and Company, Franklin Lakes, NJ, USA), following the protocol described in [60,65].

The blood was first centrifuged at 150× *g* for 15 min (Sigma 2-16kl centrifuge (Sigma Laborzentrifugen GmbH, Osterode, Germany), 12148-H (Biosafe Fixed-Angle) rotor was used) at room temperature to obtain platelet-rich plasma. This supernatant was then centrifuged at 390× *g* for 5 min, and the resulting platelet pellet was resuspended in PBS buffer (pH 7.2) and centrifuged again at 100× *g* for 5 min.

5 µL of peptide/protein was added to 150 µL of PLT suspension (platelet count, 2.8 ± 0.6 × 10^8^ PLTs/mL) for the required final peptide/protein concentration. Suspensions of both untreated and amyloid protein-treated PLTs were placed onto sterilized glass coverslips and incubated for 30 min to allow cell adhesion. The coverslips were then gently rinsed with PBS to remove non-adherent PLTs. Adhered PLTs were fixed with 1% glutaraldehyde (pH 7.4), rinsed three times with PBS, and dried under nitrogen stream prior to AFM analysis. Platelets were chemically fixed with glutaraldehyde to preserve morphological features and enable stable high-resolution imaging. It should be noted that fixation increases the absolute stiffness. Therefore, Young’s modulus values reflect relative, concentration-dependent changes between control and amyloid-treated samples, rather than absolute physiological values.

### 2.4. AFM Images

Images and force–distance curves of fixed platelets were acquired using atomic force microscope (MFP-3D, Asylum Research, Oxford Instruments, Santa Barbara, CA 93117, USA) operating in contact mode. Silicon nitride probes (type qp-Bio, Nanosensors) with a spring constant of 0.06 N/m, a resonant frequency of 16 kHz, a conical shape, and a nominal tip radius of 8 nm were employed. The maximum applied force ranged between 10 and 35 nN, depending on tip calibration and sample properties, to ensure probing the platelet membrane without causing rupture. Indentation depths were limited to <10% of the platelet height to satisfy Hertzian contact assumptions. Cells were scanned at a rate of 1 Hz, and for each sample, images were collected from an average of 5 distinct locations on the glass slide. Image acquisition was performed at a resolution of 512 × 512 pixels, enabling detailed morphological analysis, including quantification of platelet height, spread area, and average surface roughness.

AFM data analysis, including evaluation of the area (A) of the platelet central zone, also referred to as the organelle’s zone, which contains the cellular components essential for platelet function, the PLT height, and the membrane roughness (root-mean-square roughness, R_rms_), was performed using Gwyddion-2.57 and IgorPro 6.37 software. Images of cells from both untreated controls and amyloid-treated PLTs were analyzed. For statistical analysis, 15–25 platelets per experiment and per three independent donors were examined for each experimental condition (i.e., for the different concentrations of Aβ42 peptides and α-syn samples). The platelets were selected from several AFM scan fields, with an average of five fields per sample.

The membrane roughness (R_rms_) was quantified at the central membrane region of the platelets (over a 0.1 × 0.1 µm^2^ area) following preliminary surface leveling to eliminate the influence of the platelets’ spherical curvature. R_rms_ was determined using the following equation [66,67]:

(1) $$R_{\mathrm{rms}}\;=\;\sqrt{\sum_{i=1}^{N}\frac{{\left(Zi-Zn\right)}^{2}}{\left(N-1\right)}}$$

where N is the total number of points, Zi is the height of the i-th point and Zn is the average height.

Force mapping was conducted on a 32 × 32 grid, with images acquired at a scanning speed of ca. 2 s/row. Prior to measurements, the AFM tip was calibrated on a clean glass substrate using Igor Pro 6.37 software.

The Young’s modulus (E_a_) was calculated from the force–distance curves by fitting the data using Hertz model [68]:

(2) $$F(\delta )\;=\;\frac{2E_{a}tan(\alpha )}{\pi \;(1-\nu^{2})}$$

where E_a_ corresponds to the apparent Young’s modulus, α is the apical tip angle, υ is the Poisson ratio, and δ is the indentation depth.

### 2.5. Statistical Analysis

All statistical analyses were performed using OriginPro 2022 (9.9.5.167). Data are presented as means ± standard deviation (SD). One-way ANOVA followed by Tukey’s post hoc test for multiple group comparison was applied to assess the statistical significance of the observed effects on all AFM-derived parameters. A *p*-value of less than 0.05 was considered statistically significant.

## 3. Results and Discussion

### 3.1. Morphological Profile of Platelets Interacting with Amyloid Aβ42

**Figure 1:**
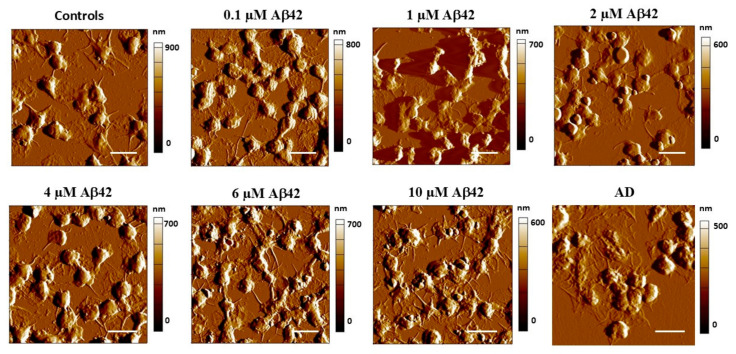
Representative AFM 2D-deflection images of untreated control and Aβ42-treated PLTs with increasing amyloid concentration. Scale bar—5 μm.

AFM images of PLTs treated with increasing concentrations of Aβ42 are presented in Figure 1. The primary effects induced by Aβ42 include PLT aggregation, pseudopodia formation, and release of alpha granules, all of which are clearly visible in the AFM images. These morphological and functional transitions during PLT activation are well-described in platelet biology literature [69,70]. PLTs’ transition through several activation states begins with a resting discoid form, characterized by a smooth surface and minimal pseudopodia. Upon stimulation, they enter an early activation state, forming filopodia, which progress to lamellipodia during the late spreading as the cytoskeleton reorganizes and integrins become activated. In the fully activated state, platelets spread extensively, expose P-selectin, and may release granule contents, promoting aggregation and thrombus formation.

In this study, the extent of PLT activation correlates directly with the concentration of Aβ42, with higher concentrations leading to a more advanced activation state. Untreated control platelets exhibit a slightly activated state, likely due to adhesion and interaction with the substrate [71]. Some filopodia are present, indicating an early activation phase, during which platelets extend these thin, actin-rich projections to explore their environment and establish initial adhesion to the substrate. No significant changes in the activation state of PLTs are observed up to 2.0 µM Aβ42. However, PLT aggregation increases, suggesting that Aβ42 at these concentrations primarily enhances cell–cell interactions rather than triggering full activation. At intermediate concentrations (2–4 µM Aβ42), platelets progress to a more activated state, as evidenced by increased cell–cell interactions and the formation of a denser network of filopodia. At the highest two Aβ42 concentrations used in this work (6 µM and 10 µM), platelets reach the fully activated state, characterized by increased cell spreading and the transition of filopodia into pseudopodia and lamellipodia—broad membrane protrusions. The presence of these structures reflects an advanced stage of cytoskeletal rearrangement and is often associated with firm adhesion and spreading of platelets on surfaces [70].

At the highest Aβ42 concentration used in this work (10.0 µM), platelets reach a procoagulant/apoptotic state, characterized by complete cell spreading and pseudopodia loss, that is close to the morphological pattern of PLTs derived from AD patients [60].

When platelets undergo activation, they extend thin filopodia outward. While the overall surface area increases, the central body, comprising cell organelles and granules, shrinks due to actin cytoskeleton reorganization, which pulls the membrane into outward extensions (filopodia) [71]. As platelet activation advances, filopodia evolve into thicker, broader pseudopodia, interspersed with lamellipodial sheets. This transition is associated with membrane contraction, leading to a reduction in the main body area as the cell spreads and flattens. The cytoskeletal rearrangement causes the platelet’s core region to become more compact, while its periphery extends dynamically.

**Figure 2:**
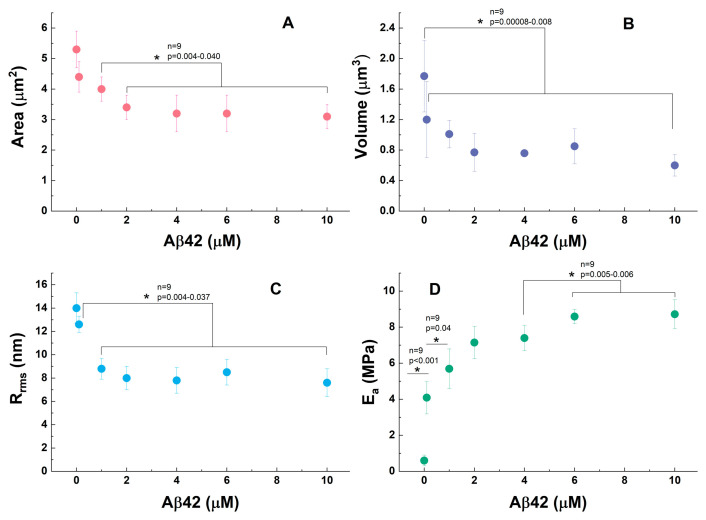
Morphometric parameters (main body area (**A**), volume (**B**), roughness (Rrms), (**C**) and Young’s modulus (E_a_) (**D**)) of PLTs for healthy control and treated with Aβ42 (0.1–10.0 µM). One-way ANOVA with Tukey’s post hoc test; * *p* < 0.05; exact *p*-values and sample sizes (n) are reported in each graph.

The morphological information provided from the AFM images of Aβ42-treated PLTs is supplemented by the values of their mean body area, volume, and membrane roughness, as well as data about the mechanical stiffness of the cell membranes (Figure 2). Both the mean area and the volume of PLTs decrease upon increasing Aβ42 concentration. The most dramatic decrease occurs at concentrations below 2.0 µM for area and below 1.0 µM for volume, while above these concentrations, the values of both the area and the volume are statistically indistinguishable (*p* < 0.05), indicating that a plateau has been reached (Figure 2A,B). The platelet area in the plateau is 1.6–1.7 times lower than the area of control PLTs (Figure 2A). The formation of filopodia and pseudopodia during platelet activation involves significant cytoskeletal reorganization, leading to morphological changes [72,73]. While these protrusions increase the overall surface area of the platelet, they can result in a reduction in the main body’s area due to membrane redistribution. The same reason underlies the decrease in the PLT volume upon the Aβ42 treatments, which follows the same trend as the main body area (Figure 2B).

**Figure 3:**
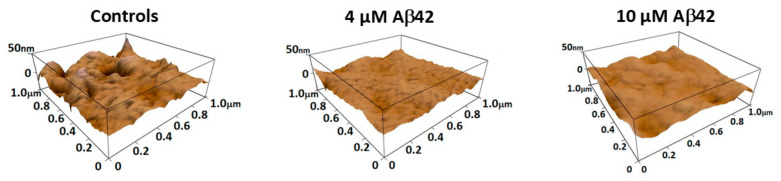
Representative AFM 3D-topographical images of the central membrane region of platelets illustrating the concentration-dependent effect of Aβ42 on membrane roughness. All images were recorded over a 1 μm × 1 μm area with a vertical scale of 50 nm.

On the other side, the formation of filopodia and pseudopodia during platelet activation can also be associated with decreased membrane roughness on the platelet’s main body, as shown in Figure 2C and Figure 3. The control sample (Figure 3 left) displays moderate roughness with a relatively uniform topography, although some surface irregularities are present.

Treatment with 4.0 μM Aβ42 (Figure 3 middle) results in a visibly smoother surface with reduced topographical features. At 10.0 μM Aβ42 (Figure 3 right), the membrane appears further flattened and homogenous, indicating a progressive decrease in surface roughness with increasing peptide concentration.

As platelets activate, spread, and form pseudopodia, their main body’s membrane roughness decreases, primarily due to cytoskeletal reorganization, redistribution of membrane components, and granule release [72]. An AFM study describes the continuous shape changes in platelets upon activation, noting that resting platelets have a wrinkled appearance due to numerous shallow folds that increase the surface area [74]. As platelets activate, they extend filopodia and pseudopodia, leading to a spread morphology with a denser central body. PLTs’ early activation can lead to a local smoothing of the main body, as the cytoskeletal rearrangement supports outward extension of thin filopodia, redistributing membrane components. During full activation, as platelets spread and pseudopodia replace filopodia, the main body flattens even more, reducing its roughness. In the late activation stage, or apoptosis, PLTs reach a fully spread morphology. Shedding of microparticles and release of granules can make the remaining membrane smoother, as many of its surface features are lost.

### 3.2. Nanomechanical Properties of Amyloid Aβ42-Treated Platelets

The Young’s modulus of platelet membranes, indicative of their stiffness, undergoes significant changes upon interaction with Aβ42 (Figure 2D). In their inactive state, platelets exhibit a relatively high Young’s modulus, reflecting a stiff membrane. Lower value of Young’s modulus was reported for the central region (around 32 kPa) and higher value for the outer region (224 kPa) of platelets, indicating a softer central and a stiffer outer region, respectively [73,75].

In this study, glutaraldehyde-fixed platelets were used for AFM measurements. We acknowledge that fixation of platelets with glutaraldehyde substantially increases their stiffness. Studies have reported that glutaraldehyde-fixed and dehydrated platelets exhibit Young’s modulus values ranging from 30 to 130 MPa, which is significantly higher than that of living, unfixed platelets [76]. Fixation was, however, necessary to stabilize platelet morphology during nanoscale force mapping, as live platelets undergo rapid activation-dependent shape changes and are highly sensitive to mechanical stimulation, which can itself trigger activation and introduce major variability in AFM recordings [77]. By fixing the samples, we ensured reproducible surface topography and minimized movement artifacts, allowing reliable comparison across experimental groups. Nevertheless, the interpretation of stiffness in terms of physiological activation must be made cautiously. Published AFM studies demonstrate that glutaraldehyde-fixed platelets exhibit stiffness values that are typically an order of magnitude higher than live platelets; therefore, the values obtained here should be understood as relative rather than absolute indicators of mechanical behavior [78]. Our approach thus enables meaningful comparison between conditions under standardized preparation, while acknowledging the limitations in translating these results directly to in vivo platelet biomechanics.

Our control PLTs, which are in an early activation state, exhibit an E_a_ of 0.6 MPa. An initial sharp eightfold increase in E_a_ is observed at 0.1 µM Aβ42, followed by a further rise at 1.0 µM Aβ42, indicating a rapid stiffening of the membrane. This significant jump suggests that, despite the morphological similarities observed in the AFM images, the activation state and cytoskeletal reorganization of the platelets advance in response to increasing Aβ42 concentrations. Representative AFM force–indentation curves for untreated PLTs and PLTs exposed to 2 µM and 6 µM Aβ42 are presented in Figure S1 (Supplementary Materials). This stiffening trend continues progressively up to 6.0–10.0 µM Aβ42, aligning well with studies demonstrating that platelet activation leads to a substantial increase in Young’s modulus compared to resting platelets [61,75]. Furthermore, another characteristic of strongly activated platelets is the variability in their Young’s modulus, which can range from 1 to 50 kPa (unfixed PLTs), depending on the specific measurement location [79].

All the observed transformations in platelets interacting with Aβ42 can be due to cytoskeletal reorganization and redistribution of membrane components. The PLT’s plasma membrane, like other biomembranes, is a complex structure made up of phospholipid bilayer [80,81]. In platelet membrane, phosphatidylcholine and sphingomyelin are located predominantly in the outer leaflet of the lipid bilayer and the polar phospholipids in the inner one, while cholesterol is asymmetrically distributed across the two leaflets [78,79,80,81,82].

In addition, platelet raft domains, i.e., macrodomains containing densely packed cholesterol, sphingolipids and gangliosides (GM1 ganglioside) [83,84,85,86] are enriched with signaling proteins involved in platelet functions and activation [87,88]. In this context, it is recognized that amyloid proteins, including Aβ42 peptide and α-syn protein, have the ability to associate with lipid membranes, which affects their pathogenicity. Studies with model lipid membranes showed strong interaction of Aβ42 with negatively charged phospholipids [89,90,91], phosphatidic acid or cardiolipin [92], as well as with lipid rafts [83,84,85,86]. Strong electrostatic interaction of the aromatic residues of soluble oligomeric Aβ42 with the positive charges of GM1 is involved in the insertion of Aβ42 into the membrane [93]. This interaction leads to conformational changes and aggregation of the peptide, as well as formation of extended β-sheets [86,94,95].

As demonstrated in the present and other studies [96,97,98,99], Aβ peptides, oligomeric and fibrillar, induce activation and aggregation of PLTs. These phenomena might be attributed to reorganization of platelet lipid rafts, which experienced changes in composition and cytoskeletal alterations, leading to a loss in the cytoskeletal proteins upon platelet activation and subsequent enrichment of the raft domains with cytoskeletal proteins [100]. This reorganization may also explain the altered platelet dimensions and membrane surface properties along with pseudopod and lamellipodia development.

### 3.3. α-Synuclein Modifies PLT Morphometry and Nanomechanics

**Figure 4:**
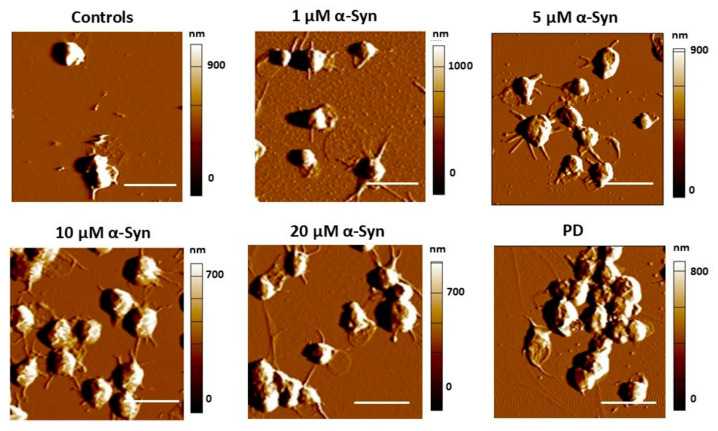
Representative AFM 2D-deflection images of untreated control and α-syn-treated PLTs with increasing protein concentration. Scale bar—5 μm.

Based on the AFM images shown in Figure 4, the morphological changes in PLTs in response to increasing α-syn concentration appear less pronounced than those triggered by Aβ42.

As already mentioned above, control untreated PLTs maintain a round, discoid shape, characteristic of resting platelets, with a smooth membrane and only minor early-stage development of short filopodia. Upon treatment with 1.0 µM α-syn, slight activation is observed, marked by the emergence of thin filopodia, suggesting an early-stage response. However, platelets largely remain separate, with no visible aggregation.

At 5.0 µM α-syn, activation becomes more evident, as longer filopodia extend outward. Some clustering and initial platelet–platelet interactions are noticeable. With further increase to 10.0 µM α-syn, activation progresses slightly, with a transition of some filopodia into broader pseudopodia and an increase in platelet aggregation. However, platelets still retain much of their original morphology. At the highest α-syn concentration (20.0 µM), platelets exhibit extensive aggregation, with membrane fusion occurring between adjacent cells. Some loss of distinct filopodia and pseudopodia is observed, and lamellipodial sheet formation becomes evident. However, despite these signs of activation, the main cell body remains largely unchanged, lacking the characteristic transition into the fully spread “fried egg” morphology typical of strongly activated or procoagulant platelets. These observations suggest that while α-syn induces some degree of platelet activation and aggregation, its effects on cytoskeletal remodeling are less dramatic compared to those caused by Aβ42, which leads to full spreading, pseudopodia dominance, and membrane restructuring.

**Figure 5:**
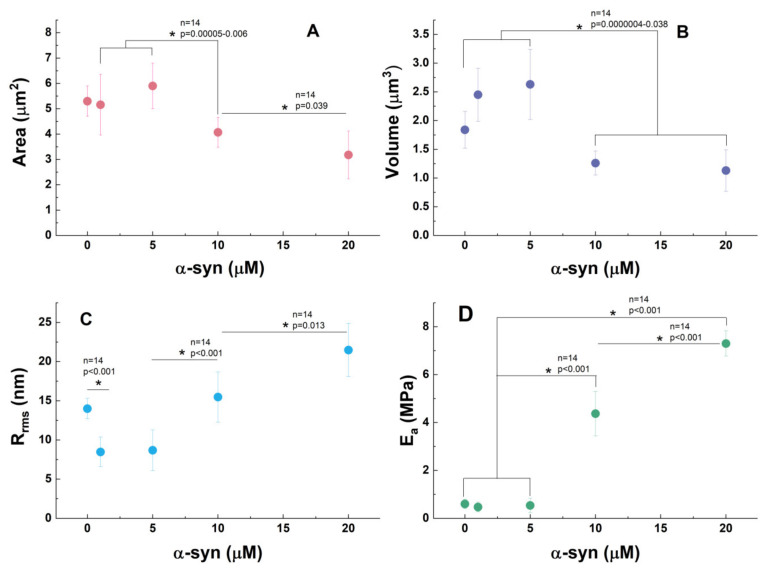
Main body area (**A**), volume (**B**), roughness (Rrms) (**C**), and Young’s modulus (E_a_) (**D**) of control untreated PLTs and PLTs treated with increasing concentrations of α-syn (1.0–20.0 µM). One-way ANOVA with Tukey’s post hoc test; * *p* < 0.05; exact *p*-values and sample sizes (n) are reported in each graph.

The variations in PLT membrane morphology and nanomechanical parameters following interaction with α-syn exhibit distinct trends compared to those induced by Aβ42 peptide. As shown in Figure 5, PLT area and volume remain statistically unchanged within the 0–5.0 µM α-syn concentration range but decrease as protein concentration increases further.

In line with these morphological changes, the Young’s modulus does not change notably within the 0–5.0 µM α-syn range but significantly increases at higher concentrations (Figure 5), indicating strong rigidification of the PLT membrane. Representative AFM force–indentation curves for untreated PLTs and PLTs exposed to 5 µM and 10 µM α-syn are presented in Figure S2 (Supplementary Materials). A similar trend is observed upon Aβ42 peptide treatment (Figure 2).

However, a key difference between the effects of Aβ42 and α-syn on PLT morphometric and nanomechanical features is that Aβ42 leads to membrane smoothing, whereas α-syn initially decreases the membrane roughness (1.0–5.0 µM) but subsequently increases it, reaching significantly higher roughness levels at 20 µM compared to control PLTs. This behavior may be attributed to the accumulation of granules beneath the membrane during platelet activation, which are not released (disturbed degranulation), leading to an increase in membrane roughness at higher α-syn concentrations [70,82,101].

**Figure 6:**
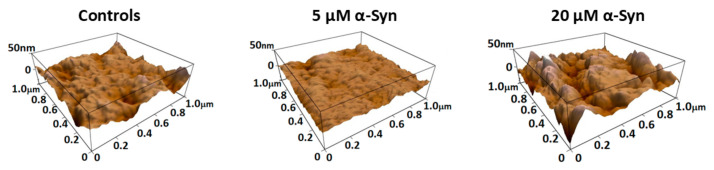
Representative AFM 3D-topographical images of the central membrane region of platelets illustrating the concentration-dependent effect of α-syn on membrane roughness. All images were recorded over a 1 μm × 1 μm area with a vertical scale of 50 nm.

AFM topographical images of the central membrane region of PLTs also illustrate the concentration-dependent effect of α-syn on membrane roughness (Figure 6). In the control sample (Figure 6 left), the membrane surface shows moderate roughness with a relatively uniform topography. Upon treatment with 5.0 μM α-syn (Figure 6 middle), the membrane becomes noticeably smoother, indicating a reduction in surface irregularities as observed for Aβ42-treated platelets. However, exposure to a higher concentration of 20.0 μM α-Syn (Figure 6, right) leads to a dramatic increase in surface roughness, with prominent protrusions and perturbations appearing across the membrane. These findings suggest that α-syn exerts a biphasic effect on platelet membrane morphology, with initial smoothing at lower concentrations followed by structural disruption at higher levels.

Several studies have explored the role of α-syn in regulating platelet granule release. Evidence suggests that α-syn may act as a negative regulator of this factor. A study demonstrated that exogenous α-syn inhibits ionomycin- or thrombin-induced α-granule secretion in human platelets in a dose-dependent manner, without affecting the release from dense or lysosomal granules [102]. An in vivo study further confirmed that α-syn functions as an inhibitor of platelet exocytosis and proposed a mechanism for this inhibitory action [103]. Additionally, research indicated that α-syn facilitates SNARE complex formation in platelets [104]. Upon activation, α-syn undergoes serine 129 phosphorylation and relocates to the platelet membrane, increasing its association with SNARE proteins such as VAMP 8, syntaxin 4, and syntaxin 11. This process is calcium- and RhoA/ROCK-dependent and can be inhibited by prostacyclin (PGI_2_) [104]. Exogenous α-syn has been found to have mild antiaggregating properties in vitro, acting as a negative regulator of platelet activation by preferentially inhibiting P-selectin expression on the platelet surface [53]. Aggregated α-syn has been shown to activate the calcium pump SERCA, leading to calcium dysregulation, which could affect platelet function [105].

α-Syn can also interact with lipid membranes, which, on the one hand, affects its conformation and, on the other, the structure and function of the lipid membrane [83,106]. Importantly, the binding interaction of α-syn with lipids is dependent on the composition and the curvature of the lipid membrane [107,108,109]; the lipid rafts are shown to facilitate the interaction [84,85,92]. The protein can bind with its positively charged N-terminus to the acidic head groups of phospholipids and adopt an α-helical conformation [89,90,91,110].

A study on platelet-rich plasma and whole blood proved that α-syn, exogenous and cytoplasmic (endogenous), binds to the outer surface of the PLT membrane, especially of activated platelets, and acts as a platelet antiaggregating protein [53]. The authors attributed the localization of α-syn on the activated PLT membrane surface to electrostatic interaction favored by exposure of negatively charged phospholipids caused by platelet activation, as shown for the binding of α-syn with its N-terminus to negatively charged model lipid membranes [53,111,112]. The highly negatively charged C-terminus, on the other hand, is responsible for α-syn interaction with proteins [111]. In addition, the hydrophobic non-amyloid β-component (NAC) region of α-syn facilitates its penetration into platelets and may regulate α-granule release [105].

Furthermore, it was estimated that activated platelets undergo a two-fold reduction in cross-sectional area and a three-fold decrease in mean volume compared to resting platelets [113]. Based on these values and assuming that all plasma α-syn binds to the surface of activated platelet membranes, Acquasaliente et al. [53] estimated an apparent local α-syn concentration of 0.93 μM. When α-syn was applied at 1.0 μM—closely matching this estimated local concentration—our data revealed a noticeable decrease in membrane roughness, while other morphological parameters remained largely unaffected. In contrast, at a concentration of 20.0 μM α-syn, all studied parameters were significantly affected (Figure 5), consistent with the expected impact of elevated local α-syn levels resulting from endogenous secretion and membrane binding on activated platelets [53].

Our results reveal that exposure of platelets to Aβ42 and α-synuclein induces distinct, concentration-dependent changes in morphology, membrane roughness, and stiffness, consistent with activation-associated remodeling. These nanomechanical alterations are nonlinear and amyloid-specific, and importantly, membrane integrity is preserved, supporting the view that the observed effects arise from targeted cytoskeletal responses rather than nonspecific membrane stress [114]. Future studies combining AFM with functional assays, such as P-selectin expression, PAC-1 binding, or thrombin-induced aggregation, will be important to validate the precise functional consequences of amyloid–platelet interactions.

Amyloid-β42 binds to platelet surface receptors such as GPVI and integrin αIIbβ_3_, triggering intracellular signaling cascades (e.g., ITAM-mediated phosphorylation of FcγRIIA, PLC, PKC, and PI3K) and reactive oxygen species (ROS) production, which collectively promote platelet activation and aggregation [99,100,115]. These signaling events promote actin polymerization, myosin contractility, and membrane spreading, consistent with the progressive transition from filopodia to pseudopodia and lamellipodia observed in our AFM images (Figure 1). The rapid stiffening of the membrane (increased E_a_) at low Aβ42 concentrations likely reflects an early cytoskeletal tension associated with GPVI- and αIIbβ_3_-mediated activation [116]. These interactions also alter cytoskeletal organization and support amyloid clustering on the platelet membrane, contributing to vessel-associated amyloid formation [48].

In contrast, α-synuclein modifies platelet function through partially distinct mechanisms. In the case of α-synuclein, the protein localizes to the surface of activated platelets and can modulate platelet function by interacting with the SNARE complex. This regulation affects granule secretion and cytoskeletal dynamics, thereby influencing platelet activation states [103]. This regulatory role explains the biphasic changes in membrane roughness, i.e., initial smoothing at lower α-syn concentrations is consistent with mild activation and limited degranulation, whereas increased roughness at higher concentrations likely reflects disturbed α-granule release and membrane protrusion associated with impaired vesicle fusion. The comparatively weaker effects of α-syn on morphology and stiffness indicate that its primary influence lies in modulating secretory machinery rather than driving full cytoskeletal interaction. At high α-syn concentrations, the marked increase in E_a_ may reflect a tension-like response, possibly due to calcium dysregulation [117].

Taken together, the distinct nanomechanical signatures of platelets interacting with Aβ42 and α-syn (Aβ42-driven uniform stiffening and membrane smoothing versus α-syn-dependent biphasic roughness and delayed stiffening) likely reflect their divergent modes of receptor engagement, cytoskeletal activation, and granule regulatory functions. These alterations are expected to translate into functional differences in adhesion, aggregation, and procoagulant activity in circulating cells. Importantly, exposure of healthy platelets to these amyloids reproduces key morphological and mechanical features observed in patient-derived platelets, supporting the notion that circulating amyloid peptides directly influence platelet biophysics and may contribute to disease-associated vascular or hemostatic alterations. These findings extend beyond our previous study by revealing how different amyloid species differentially modulate platelet nanomechanics and morphology, thereby refining our understanding of platelet involvement in neurodegenerative diseases.

## 4. Conclusions

Using AFM in both imaging and force–distance modes, we provide a comprehensive picture of the morphological and mechanical transformation of platelet membranes from the resting to activated state, induced by interaction with amyloid Aβ42 peptide and α-syn protein. Platelet activation and cytoskeletal reorganization progressed in a concentration-dependent manner. This process culminated in a fully activated state, manifested by increased spreading, filopodia transition into pseudopodia, and lamellipodia upon interaction with Aβ42. These cytoskeletal rearrangements were accompanied by a remodeling of membrane architecture, from a smooth membrane with a minimal early-stage development of short filopodia in resting platelets to a compact central core region with dynamic peripheral extensions in activated platelets.

Aβ42 induced a progressive decrease in platelet membrane roughness, indicating membrane smoothening, while α-syn led to a significant increase in roughness, likely due to impaired granule release.

The most pronounced effect of both Aβ42 and α-syn was observed in membrane stiffness: increased stiffness was closely associated with a reduction in platelet spreading, suggesting a functional link between mechanical properties and morphological changes.

Importantly, our findings support the hypothesis that the altered morphological and nanomechanical signatures of platelets observed in neurodegenerative disorders such as Parkinson’s and Alzheimer’s diseases may be, at least in part, attributed to the direct interaction of platelet membranes with amyloid proteins.

## Data Availability

Data supporting the conclusions of this article will be made available by the corresponding authors on request.

## Supplementary Materials

The following supporting information can be downloaded at https://www.mdpi.com/article/10.3390/biomedicines13123104/s1, Figure S1: Representative force–indentation curves obtained from PLT samples of untreated cells (A), and cells treated with 2 µM (B) and 6 µM (C) Aβ42; Figure S2: Representative force–indentation curves obtained from PLTs samples of untreated cells (A), and cells treated with 5 µM (B) and 10 µM (C) α-syn.
