# Supplementary material for: Amyloid Protein-Induced Remodeling of Morphometry and Nanomechanics in Human Platelets
Source: Biomedicines. 2025 Dec 16;13(12):3104. doi: 10.3390/biomedicines13123104 (PMC12730995; doi:10.3390/biomedicines13123104)
Supplement: Supplementary file 1 [file biomedicines-13-03104-s001.zip › biomedicines-4031399-supplementary.pdf]

Amyloid proteins-induced remodeling of morphometry and nanomechanics in human platelets

Tonya D. Andreeva[1,2†*], Svetla Todinova[†2], Ariana Langari[2], Velichka Strijkova[3], Vesela Katrova[3], and Stefka G. Taneva[2,*]

[1] Faculty Life Sciences, Reutlingen University, Alteburgstraße 150, 72762 Reutlingen, Germany; tonya.andreeva@reutlingen-university.de (T.A.)

[2] Institute of Biophysics and Biomedical Engineering, Bulgarian Academy of Sciences, "Acad. G. Bonchev" Str. 21, 1113 Sofia, Bulgaria; tonya.andreeva@reutlingen-university.de (T.A.); todinova@abv.bg (S.T); arianalangari@abv.bg (A.L.); sgtaneva@gmail.com (S.G.T.)

[3] Institute of Optical Materials and Technologies "Acad. Yordan Malinovski", Bulgarian Academy of Sciences, "Acad. G. Bonchev" Str. 109, 1113 Sofia, Bulgaria; vily@iomt.bas.bg (V.S.); vesela.lozanova@abv.bg (V.K.)

[†] These authors contributed equally to this work

* tonya.andreeva@reutlingen-university.de (T.A.); sgtaneva@gmail.com (S.G.T.)

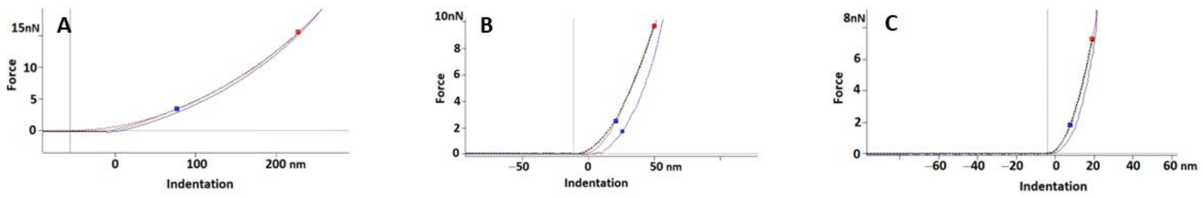

Figure S1. Representative force–indentation curves obtained from untreated PLTs (A), and PLTs treated with 2 µM (B) and 6 µM (C) Aβ42 .

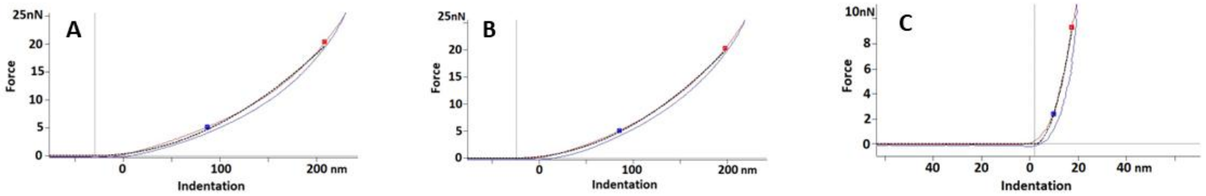

Figure S2. Representative force–indentation curves obtained from untreated PLTs (A), and PLTs treated with 5 µM (B) and 10 µM (C) α–syn.
